# Ras family signaling pathway in immunopathogenesis of inflammatory rheumatic diseases

**DOI:** 10.3389/fimmu.2023.1151246

**Published:** 2023-05-15

**Authors:** Mina Sadeghi Shaker, Mohsen Rokni, Mahdi Mahmoudi, Elham Farhadi

**Affiliations:** ^1^ Department of Immunology, School of Medicine, Tehran University of Medical Sciences, Tehran, Iran; ^2^ Rheumatology Research Center, Tehran University of Medical Sciences, Tehran, Iran; ^3^ Department of Immunology, University of Social Welfare and Rehabilitation Sciences, Tehran, Iran; ^4^ Inflammation Research Center, Tehran University of Medical Sciences, Tehran, Iran

**Keywords:** autoimmune inflammatory diseases, Ras signaling, rheumatoid arthritis, systemic lupus erythematosus, systemic sclerosis, ankylosing spondylitis

## Abstract

The Ras (rat sarcoma virus) is a GTP-binding protein that is considered one of the important members of the Ras-GTPase superfamily. The Ras involves several pathways in the cell that include proliferation, migration, survival, differentiation, and fibrosis. Abnormalities in the expression level and activation of the Ras family signaling pathway and its downstream kinases such as Raf/MEK/ERK1-2 contribute to the pathogenic mechanisms of rheumatic diseases including immune system dysregulation, inflammation, and fibrosis in systemic sclerosis (SSc); destruction and inflammation of synovial tissue in rheumatoid arthritis (RA); and autoantibody production and immune complexes formation in systemic lupus erythematosus (SLE); and enhance osteoblast differentiation and ossification during skeletal formation in ankylosing spondylitis (AS). In this review, the basic biology, signaling of Ras, and abnormalities in this pathway in rheumatic diseases including SSc, RA, AS, and SLE will be discussed.

## Introduction

The Ras (rat sarcoma virus)-GTPase superfamily consists of GTP-binding proteins with a low molecular weight that convert extracellular signals to various cellular functions. This superfamily is sub-classified into families and subfamilies based on their members’ sequence and functional resemblances. Key members of the Ras family such as H-Ras, N-Ras, and K-Ras have an important role in the genes’ expression involved in proliferation, survival, differentiation, and fibrosis by activating transcription factors such as c-FOS, c-JUN, and ETS domain transcription factor ELK1 (1). Dysfunction or mutation of Ras family proto-oncogenes leads to different kinds of malignancies and autoimmune diseases (2). In autoimmune lymphoproliferative syndrome (ALPS), which is the most prevalent genetic disease related to defective apoptosis of lymphocytes, mutations in N-Ras lead to a reduction in Bim (a pro-apoptotic protein), which results in decreased mitochondrial apoptosis and lymphocyte accumulation (3). Recently, the role of Ras family proteins and their downstream kinases have indicated in the pathogenesis of different auto-inflammatory rheumatic diseases [systemic sclerosis (SSc), rheumatoid arthritis (RA), ankylosing spondylitis (AS), and systemic lupus erythematosus (SLE)] that targeting and inhibiting important components of this signaling cascade may be a promising approach to treat these diseases.

Based on recent evidence in fibrotic diseases, fibrosis may correlate with the stabilization of Ha-Ras by growth factors and autoantibodies, which leads to the increased expression of genes involved in the fibrosis process such as type I collagen, fibronectin, and production of ROS that has a significant role in this process (4). Abnormalities in the level of p-ERK (phosphorylated extracellular signal-regulated kinase) and its upstream kinases have been observed in some auto-inflammatory rheumatic diseases that are related to T-cell low stimulation threshold and result in the production of autoantibodies in RA patients (5). Moreover, defects in the Ras signaling pathway including GEFs (guanine nucleotide exchange factors) and ERK1/2 related to epigenetic abnormalities result in lupus-like autoimmunity (6). Osteoclastogenesis, which is abnormal in RA, is controlled by numerous interacting signaling cascades. The Ras-Raf-MEK1/2-ERK1/2 activation may function as a main key driver of human osteoclast differentiation (7). In addition, some studies have demonstrated that the ERK1/2 activity, an essential downstream mediator of Ras, enhances osteoblast differentiation and ossification in MSCs during skeletal formation (8, 9). This review will focus on the basic biology of Ras signaling and defects of Ras signaling pathway molecules in each auto-inflammatory rheumatic diseases.

## Ras family signaling pathway

The Ras molecule is a membrane-bound protein with a low molecular weight that has a GDP/GTP-bound domain. Following external stimulus by the binding of ligands including different growth factors to their receptors, signal transduction by Ras proteins takes place through reversible binding of GTP, whereas the passive form is connected to GDP. Three different kinds of protein modulator agents are responsible for the regulation of exchanging among these two states; guanine nucleotide exchange factors (GEFs) including SOS (son of sevenless class) and RasGRP1/3 (Ras guanyl releasing protein1/3) catalyze the switch from GDP to GTP to induce Ras activation while GTPase-activating proteins (GAPs) inactivate the Ras protein by hydrolysis of Ras-bound GTP to GDP. The deactivation process is also performed by guanine nucleotide dissociation inhibitors (GDIs), which are connected to the GDP-bound state and not only inhibit the switch but also prevent membrane association. Different upstream signals that activate or deactivate Ras signaling affect all of these regulatory proteins. There is a conformational change that leads to the shift of Ras molecules between the GDP- and GTP-bound states, which highly increases their affinity for downstream effectors (10).

## Ras molecule effectors

The most well-known downstream effector for Ras protein is the Raf-MEK-ERK1/2 pathway. When growth factors are bound to their receptors, phosphorylation and activation of receptors occur such that, initially, GRB2 (growth-factor-receptor-bound protein 2), an adaptor protein, is bound to the receptor through the SH2 domain and then by the SH3 domain bound to SOS. The SOS exchange GDP-bound Ras to GTP-bound Ras and then serine/threonine kinases including B-Raf and C-Raf are recruited to the plasma membrane and dimerized and activated by Ras-GTP (11). Localization of Raf protein to the plasma membrane is necessary for its activation (12, 13). Phosphorylated Raf stimulates and phosphorylates mitogen-activated protein kinase kinase 1 and 2 (MAPKK, MEK1/2) and then MEK1/2 activates mitogen-activated protein kinase (MAPK, ERK1/2). Activated ERK1/2 phosphorylates several nuclear transcription factors including ETS, ELK, c-JUN, AP1, and c-FOS, which switches on several genes related to differentiation, proliferation, and fibrosis (14).

## Ras signaling in immune cells

Ras superfamily GTPases act as a major checkpoint linkage in antigen receptors, growth mediators, interleukins, and stimulation of chemokine to immune response (15). The Ras and Rho superfamily play important roles in the activation of stromal and immune cells during an inflammatory response, and growing evidence demonstrates that changes in small GTPase signaling (Ras and Rho) promote the pathological actions of these cells populations in human persistent inflammatory diseases (16). MEK/ERKs cascade is essential for the differentiation of immune system cells. It has been indicated that MEK/ERKs activity plays a major role in granulocyte/macrophage (GM) lineage differentiation (dendritic cells and macrophages) from hematopoietic stem cells (HSCs) and common lymphoid progenitors (CLPs) (17).

Macrophages contribute to autoimmune inflammatory diseases and inflammation through their ability to present auto-antigens, disturbance of the balance between M1 and M2 macrophage phenotype, regulation of inflammatory responses, and incomplete clearance of dying cells. In general, monocyte or macrophage infiltration and change in their frequency are observed in many autoimmune diseases (18, 19).

Macrophages can change their functional phenotype to the M1 phenotype (pro-inflammatory) or the M2 phenotype (anti-inflammatory) in response to the pathogenic microbes and microenvironment. Macrophages are activated by cytokines such as transforming growth factor-β (TGF-β), IFN-γ, and TNF-α, and through SMAD-independent pathways such as Ras/MAPK/ERK and show the M1 phenotype (20). Furthermore, the Ras signaling pathway also has a role in the development of the M2 phenotype. The M2 macrophages under the influence of IL-10, IL-13, and IL-4 produce TGF-β and IL-10 that are involved in fibrosis (20, 21). Thus, under the influence of cytokines in the microenvironment, the Ras activity can promote both M1 and M2 phenotypes.

Dendritic cells are a group of small cells in the hematopoietic systems that have a main role in antigen presentation and innate immunity. Ras signaling is essential for DC development. During monocyte-derived DC (moDC) maturation, Raf kinases are stabilized and contributed to the differentiation and activation of moDCs. Furthermore, the ERK1/2 activation during DC maturation promotes inflammatory cytokine secretion such as IL-1β and TNF-α (22). Pharmacologic inhibition of MEK or ERK1/2 abolishes both differentiation and survival during moDC development (23). In addition, it has been shown that R-Ras knockout can contribute to the reduction of natural regulatory T lymphocytes, inhibition of tolerogenic DCs development, and enhancement of autoimmunity (24, 25).

The adaptive immune responses against pathogens are controlled by B cells and T cells. T helper cells (T CD4^+^) contribute to antigen exclusion by activating various cells, including macrophages, whereas T cytotoxic cells (T CD8^+^) promote cell death. Central tolerance occurs in the thymus by positive and negative selection and proper maturation of thymocytes. In the periphery, T-cell responses are also controlled through the activation of anergy or regulatory T cells (26). The Ras-ERK1/2 activity has a major role in the positive selection, maturation of thymocytes, and differentiation toward CD8^+^ or CD4^+^ T cells (27). Antigen recognition through the TCR receptor gives rise to the PKC-γ signaling activation and Ras-ERK pathway, which results in the activation of transcription factors including JUN, AP-1, and c-FOS that contribute to T-cell activation and induction of cytokine genes including IL-2 and TNF-α (26, 28). Moreover, the Ras-ERK1/2 signaling pathway increases IL-4 overexpression in T cells (29), which is known as the main cytokine in fibrosis and wound healing. In addition, it has been reported that decreased RasGRP1 expression, a critical regulator in lymphocyte receptor signaling, results in the expansion of impaired T lymphocytes in mice (C57BL/6) and inflammation in autoimmune diseases (30).

The Ras signaling pathway is also activated downstream of the B-cell receptor (BCR) and plays a major role in the survival, differentiation, and function of B lymphocytes (31, 32). Furthermore, the Ras-ERK1/2 pathway helps Th2 lymphocyte differentiation and follows B lymphocyte activation (33). Ras signaling in B lymphocytes is activated by BCR signaling and sarcoma family kinases (Src kinase)/Syk pathway, and increased expression of Ras results in loss of tolerance for both central and peripheral B lymphocytes and causes autoantibody production, tissue damage, and fibrosis (34, 35). Furthermore, the MEK-ERK1/2 activation is essential in upregulating BAFFR and breaking tolerance in central and peripheral B lymphocytes (36). Ras pathway persistent stimulation in autoreactive B cells results in the prevention of receptor editing, cell differentiation, and IgG autoantibody production. An overstimulated form of Raf gives rise to a lower κ-to-λ light chain ratio in mice, suggesting that the Ras-Raf-ERK1/2 activity cascade prevents receptor editing. Phosphorylated ERK1/2 levels are commonly higher in non-auto-reactive than auto-reactive immature B lymphocytes (34). Ras defect may decrease the survival of pre-B cell, and this issue could support the idea that Ras defect leads to B-cell survival deficiency. The prevention of Ras activity results in a 10-fold diminution in the abundance of transitional B cells (T1, T2, and T3), likely reflecting a developmental delay at the transition from pro-B to pre-B cells. BCR activity through the Ras-Raf-MEK signaling pathway contributes to the ultimate differentiation of IgG memory B cells into Ig-secreting plasma cells. The Ras-Raf-MEK cascade in BCR signaling prevents apoptosis of memory B cells during plasmacytic differentiation (37). In addition, RasGRP1 and RasGRP3 were indicated to regulate the activation of Ras and the ERK1/2-MAP kinase pathway (38), such that RasGRP3 is responsible for the initial level of Ras-GTP expression in the non-stimulated B lymphocytes and its diminished expression leads to deficiency in antibody (Ab) production and hypo-gammaglobulinemia, but reduced expression of RasGRP1 results in autoimmunity and production of antinuclear Abs (ANA) due to defects in the function of T lymphocytes (39).

Thus, changes in the Ras activity can lead to alteration in B-cell selection and development with the potential to affect the improvement of autoimmunity. Therefore, Ras activation can alter the selection pattern of autoreactive cells, blocking immunoglobulin gene rearrangement *via* PI3K, promoting cell differentiation *via* ERK1/2, and leading to the production of autoantibodies and the preservation of memory B cells.

## Ras signaling in the EMT process and fibrosis

The Ras family signaling has a role in the fibrosis process by promoting the epithelial-mesenchymal transition (EMT), increasing fibroblast cell proliferation, and involving in growth factors signaling which among them, TGF-β, PDGF, and IL-6 are the main (40). During the EMT, an epithelial cell phenotype changes to a mesenchymal cell such as a myofibroblast in the presence of specific growth factors, especially TGF-β (41). Some studies implicated that high expression of Ras promotes EMT in response to TGF-β by the upregulation of leukotriene B4 receptor-2 (BLTR2) that activates downstream factors including ROS and NF-κB, which have a major role in EMT (42). The H-Ras and ki-Ras isoforms regulate the extracellular matrix (ECM) expression, proliferation, and migration of fibroblast cells (40, 43). Different growth mediators including EGF, FGF, HGF, VEGF, and PDGF activate the Ras-Raf-MEK-ERK1/2 signaling cascade that contributes to EMT. The Ras-Raf-MEK-ERK1/2 signaling activation leads to enhanced expression of EMT-activating transcription factors including snail1/2, zinc finger E-box-binding homeobox (Zeb1/2), and Twist1/2, which contribute to increased mesenchymal proteins and repress epithelial proteins expression (44).

Moreover, ERK1/2 signaling, which is located in Ras downstream, regulates the EMT process by reducing adherens junctions such as E-cadherin, actin stress fibers induction, and cell motility in the presence of TGF (α and β), PDGF, and IL-6 (45–47).

**Figure 1:**
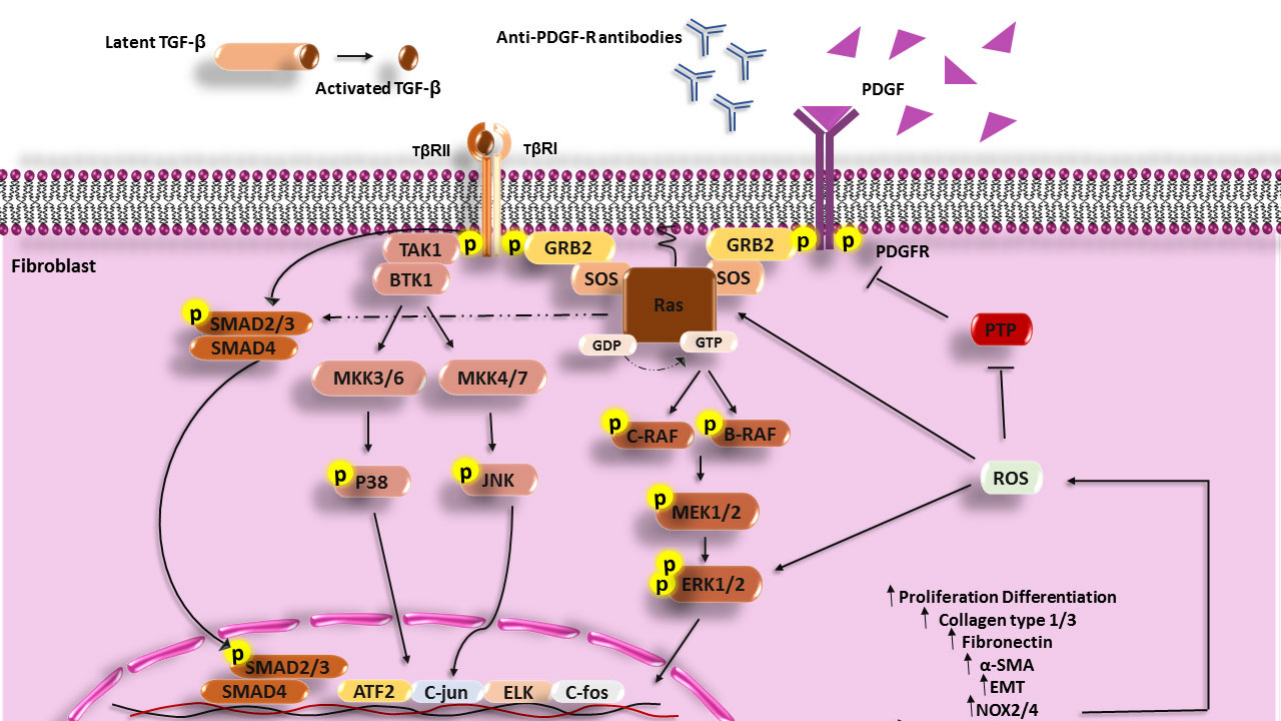
Scheme of the Ras signaling pathway that increases in the fibrosis process and SSc disease. PDGF, Anti-PDGFR antibodies can activate MAPK signaling in fibroblasts of SSc patients and cause expression enhancement of involved genes in the fibrosis process and also TGF-β can through both the Smad pathway and non-Smad pathway (PI3k, JNK, and P38) activate fibroblasts, increase the synthesis of collagen, fibronectin and develop fibrosis. TGF-β, Transforming growth factor beta; TβR, Transforming growth factor beta receptor; PDGF, Platelet-derived growth factor; PDGFR, Platelet-derived growth factor receptor; GRB2, Growth factor receptor-bound protein 2; MEK, Mitogen-activated protein kinase kinase; ERK, extracellular signal-regulated kinase; PIP2, Phosphatidylinositol 4; 5-bisphosphate; PIP3, Phosphatidylinositol (3; 4; 5)-triphosphate; PI3K, Phosphoinositide 3-kinases; PTEN, Phosphatase and tensin homolog; TAK1, TGF‐β‐activated kinase 1; MKK, The mitogen-activated protein kinase kinase; PTP, Protein tyrosine phosphatases; NF-κB, Nuclear Factor kappa-light-chain-enhancer of activated B cells; ROS, Reactive oxygen species; ATF2, Activating transcription factor 2.

TGF-β1 through stimulation of both SMAD (small mothers against decapentaplegic homolog [canonical pathway]) and non-SMAD (non-canonical pathway) signaling pathways contributes to fibrosis. In the canonical pathway, activated TGF-β1 acts through SMAD molecules. This cytokine also activates non-SMAD signaling including PI3K, Rho GTPase, and MAPK (48, 49). Stimulation of human skin fibroblasts by TGF-β1 results in H-Ras activation and ERK1/2, which causes the enhanced production of collagen type I, fibronectin (FN), smooth muscle alpha-actin (α-SMA), and ROS, and differentiation of fibroblasts and other cells (epithelial and endothelial cells) to myofibroblasts (50). Furthermore, increased H-Ras protein levels motivate SMAD2/3 signaling and the expression of collagen I independently of stimulation with TGF-β (4) (**Figure 1**).

TGF-β1 can induce isoforms of NADPH oxidase (NOX4) and produce a large amount of ROS that has an important role in fibrosis. Thus, protein tyrosine phosphatases (PTPs) and/or phosphatase and tensin homolog (PTEN) are inhibited under the influence of excessive oxidative activation. Overproduction of ROS leads to the activation and stabilization of tyrosine kinases including ERK1/2 and Ras, MAPKs, JNK, PKC, PI3K, and transcription factors which are involved in fibrosis (51–55). In addition, K-Ras promotes ROS generation *via* the upregulation of NOX1, which is an essential regulator for the K-Ras (56, 57). Inhibition of ROS production by using NOX1 and NOX4 inhibitors (GKT137831) has revealed that ROS has a crucial role in the production of collagen I, FN, and the expression of α-SMA (54).

Another characterized stimulator for fibrosis is the platelet-derived growth factor (PDGF) (58). PDGF through the H-Ras signaling pathway and phosphorylating different MAP kinases cascade (Raf-MEK1/2-ERK1/2) activates transcription factors such as ELK, c-FOS, and c-JUN, and these transcription mediators increase the overexpression of involved genes in the fibrosis (in addition to promoting myofibroblast differentiation and ECM production especially collagen type I and FN) (59). PDGF contributes to the enhancement of ROS production through the induction of NOX2/NOX4 and the development of fibrosis (60). Some investigations have defined a substitute mechanism for PDGFR activation through the binding of autoantibodies to PDGFRs. Following IgG binding to the PDGFRs and the stimulation of PDGFR, downstream signaling molecules such as Ras-Raf-MEK1/2 and ERK1/2 are activated and induce genes involved in fibrosis and ROS by activating the NADPH oxidase complex (59).

Furthermore, ROS through a positive feedback mechanism induces increased expression of H-Ras and activates ERK1/2 signaling, which has a critical role in fibrosis in the presence of PDGF. In addition, it has been found that upon stimulation of healthy fibroblasts with PDGF, the level of H-Ras protein is increased (56), and fibroblast treatment with nonspecific ROS scavenger (NAC) or NADPH oxidase inhibitor (diphenyleneiodonium [DPI]) has shown that ROS contributes to both H-Ras induction and ERK1/2 activation by PDGF (56). Thus, TGF-β1, and PDGF contribute to EMT and fibrosis through activation of the Ras signaling pathway and ROS production.

## Ras signaling in inflammation and induction of adhesion molecules

New studies have implicated that overexpression of Ras protein increases inflammatory cytokine secretion such as IL-6 and IL-8 (61). It has been shown that activation of Ras signaling causes an increase in the secretion of various cytokines and chemokines such as C-X-C motif chemokine 6 (CXCL6), CXCL5, C-C motif chemokine 20 (CCL20), macrophage colony-stimulating factor (M-CSF), and insulin-like growth factor-binding protein-1 (IGFBP-1) and IGFBP-4 in mesenchymal stem cells (MSCs), which may affect the differentiation and migration of MSCs and other immune cells (62). Furthermore, inhibition of Ras signaling decreases the levels of various inflammatory interleukins, and genes relevant to inflammation, immune system, and autoimmunity (such as IL-17A/F, IL-22, IFN-γ, CSF2, LTA, and IL-1A) in effector CD4^+^ T cells (63).

Furthermore, binding of cytokines such as IL-4, IL-2, IL-3, IL-7, IL-6, IL-10, IL-11, and IFNs to their specific receptors could promote activation of different signaling cascades including the JAK-STAT, MAPK (Ras-ERK1/2), Src/ZAP70, PI3K, and other cascades (64). After cytokine binds to its receptor, the Shc adaptor protein attaches to phosphorylated tyrosine in the cytoplasmic domain of cytokine receptors and recruits Grb2 and SOS, which leads to the activation of the Ras pathway (64). Integrin, which facilitates cell–cell and cell–ECM adhesion, plays a role in inflammation through the adjustment of migration and cell adhesion. Interestingly, Ras proteins have an important role in suppressing or activating integrins (the affinity and avidity of integrins). Raf1 and ERK1/2-MAPK might mediate the prevention of integrin activation by Ras under the determined conditions, whereas Ras-dependent activation of PI3K can activate integrins (65). In addition, suppression of Ha-Ras and c-Raf expression inhibits the E-selectin overexpression and vascular adhesion molecule-1 (VCAM-1 or CD106) induced by TNF-α (66). Thus, the Ras signaling pathway and molecules involved in this pathway enhance inflammation, especially through the induction of adhesion molecules.

## Ras signaling in osteoblast and osteoclast differentiation

Different stimuli especially receptor activator of nuclear factor-kappa B ligand (RANKL), M-CSF, interleukin-1β (IL-1β), IL-6, and IL-34 affect monocyte precursors and increase the Ras-Raf interaction and follow the stimulation of the MEK1/2-ERK1/2 signaling cascade, which contributes to the migration, survival, and differentiation of monocyte precursors to pre-osteoclasts (67, 68). Furthermore, it has been revealed that blockage of MAPK protein expression (ERK1/2 and JNK) can inhibit osteoclast differentiation (69). Mononuclear pre-osteoclasts in the presence of GM-CSF through the Ras-ERK1/2 pathway augment c-FOS, nuclear factor of activated T cells (NFAT-c1) proteins, and dendritic cell-specific trans-membrane protein (DC-STAMP) that cause proliferation, survival, perfusion of pre-osteoclasts, and formation of activated and multinucleated osteoclasts, which can destroy bones (68, 70). M-CSF treatment of mature osteoclasts upregulated Ras, and inhibition of Ras enhances osteoclast apoptosis (71). Also, M-CSF and RANKL increase the proliferation of osteoclast precursors and the activity of osteoclasts through the PI3K/Akt cascade. Inhibition of PI3K suppresses the activation and differentiation of rodent osteoclasts. Controversially, p38 inhibition results in increased osteoclastogenesis, which is related to increased phosphorylation of ERK1/2 (67). Thus, ERK1/2 activation may play a critical role in osteoclast differentiation.

There are many controversies regarding Ras signaling in ossification. For example, lines of evidence from pharmacological therapy indicate that increased Ras-MAPK signaling may antagonize osteogenesis in a certain way, and suppression of Ras-MAPK activity *via* the MEK (U0126 and PD98059) or Ras inhibitor (salirasib) has been found to increase osteogenic activation and differentiation in cultured osteoblasts and myoblasts treated with bone morphogenic proteins (BMPs). BMPs are part of the TGF-β superfamily that, through phosphorylation and nucleolar translocation of Smads1/5/8, promote osteogenic gene expression. Therefore, potential mechanisms related to this impact are the phosphorylation of Smads and the inhibition of their nuclear import by ERKs. In contrast, some reports reinforce a conflicting idea that supports an osteogenic role of Ras-MAPK activity. The Ras-ERK1/2 signaling can augment phosphorylation and activity of transcription factor Runx2, or may directly affect the expression of the osteogenic genes and contribute to bone formation. According to the promotion of Runx2 by the Ras signaling, it seems that activation of this pathway can commit MSCs to osteoblasts, but phosphorylation of Smad molecules by the Ras-MAPK pathway inhibits the final stages of osteoblast differentiation (72). Generally, this literature review concluded that Ras-MAPK activity subtly regulates osteolysis more than ossification, but it can be different depending on the type of cells and secretion mediators in the microenvironment of diseases.

## Ras family signaling in systemic sclerosis

SSc is known as a severe autoimmune and connective tissue disease that is distinguished *via* inflammation, abnormalities of the immune system, vasculopathy, and fibrosis of the dermis and several internal organs (15, 73). In genetically predisposed individuals, many stimuli include microbial agents (such as CMV), environmental agents [like vinyl chloride, silica, and reactive oxygen species (ROS)], and anti-endothelial cell antibodies (AECAs) that can cause endothelium damage and increase vascular permeability (15, 74, 75). In the dermis of SSc patients, accumulation of immune cells including macrophages, B and T cells, and DCs have been reported, which produce different kinds of pro-inflammatory (TNF-α, IL-1, IL-6, and IL-2) and pro-fibrotic (IL-4, IL-13, IL-6, and TGF-β) cytokines (76) and different autoantibodies (77, 78). In the early stages of SSc, numerous Th17 cells and enhancer levels of IL-17 have been found (79), whereas, in late stages, Th2 cells are dominant T lymphocytes of peripheral blood and skin biopsies that secrete TGF-β, IL-4, and IL-13 (80–82). An augmented level of IL-6 has been indicated in SSc that is related to the rate of fibrosis (15, 83). IL-6 increases ECM production and collagen type I expression through Ras-ERK1/2 signaling in dermis fibroblasts of SSc patients and promotes differentiation of cardiac fibroblast to myofibroblast (84). With regard to the enhanced expression of these cytokines in SSc and the activation of the Ras pathway through these cytokines, it seems that Ras activation can link the growth mediators and cytokines to fibrosis and EMT (**Figure 1**).

The serum of SSc patients is rich in cytokines and profibrotic growth factors especially PDGF and TGF-β, which have a main role in fibrosis. TGF-β and PDGF can induce ROS production and ROS can increase H-Ras protein levels through ERK1/2. Excess production of ROS and activated Ras-ERK1/2 has been reported in SSc fibroblasts. High ROS and H-Ras, and activation of ERK1/2 stimulated collagen synthesis and DNA damage, and increased senescence. Conversely, inhibition of ROS, Ras, or ERK1/2 restores the normal phenotype in SSc fibroblasts (56). Considering the role of TGF-β and PDGF in the human skin fibroblasts’ activation through non-SMAD signaling and induction of collagen type I, FN, α-SMA, and ROS production; the differentiation of different cells including fibroblasts and epithelial and endothelial cells to myofibroblasts (50); and the role of Ras/ERK signaling in ROS production and induction of fibrosis and EMT, it seems that TGF-β and PDGF act in SSc pathogenesis partly through activation of the Ras/ERK pathway.

Given the presence of different autoantibodies in SSc and chronic activation and overexpression of the Ras signaling in B cells, which results in the prevention of receptor editing, loss of tolerance in B lymphocytes, and autoantibody production (42), it seems that Ras signaling activation is correlated to autoantibody production. Moreover, TGF-β and PDGF can trigger multiple kinase proteins including the Ras-ERK1/2 activity that acts as the essential factor for developing SSc.

## Ras family signaling in ankylosing spondylitis

AS is a chronic autoinflammatory disease that primarily affects the axial skeleton. Pathological novel bone formation (ossification) is the main feature of AS. The affected joint will be immobilized when the osteophytes bridge the overall articulation cavity, leading to stiffness in axial articulation, spinal ankyloses, and permanent disability (85). The cytokines released by T and B lymphocytes contribute to the osteogenesis and pathogenesis of AS in such a way that there is a remarkable enhancement in the population of Th22 and Th17 cells as well as IL-22 in AS patients. IL-22 production by Th17 and Th22 cells is associated with IL-23, which is elevated in AS patients (86). An *in vivo* study clarifies that Ras blockers forcefully mitigate the upregulation of serum IL-22 and also the gene expression and release of IL-22 by purified effector CD4^+^ Th17 and Th22 cells. Thus, it seems that the Ras signaling activity contributes to the induction of IL-22 (63), which is overexpressed in AS patients and can promote bone formation and mineralization by metabolic change and increased glycolysis (87).

It has been indicated that Ras activation can augment ossification *in vitro* and predominant negative Ras and MAPK suppressors were also found to have anti-ossification effects. Some researchers suggested that the MAPK signaling pathway directly motivates ossification *via* the upregulation and phosphorylation of the transcription factor Runx2 through ERK2 activation (72). Similarly, global gene expression studies stated that an inflammatory microenvironment may increase the MAPK activity and M-Ras (understudied member of the Ras family) signaling pathways in MSCs isolated from AS patients and cause an increment of inflammatory gene expression (88). M-Ras is upregulated in the osteoblast and hypertrophic chondrocyte cells. Furthermore, M-Ras is overexpressed in MSCs during maturation and differentiation into osteoblasts. BMP-2 can phosphorylate M-Ras and mediate osteoblastic differentiation and maturation by p38 MAPK, Rac1, and JNK activation (89). Changes in the gene expression of Ras family members in AS MSCs include (1) increased expression of Rac1 and MAPK, which is activated by Ras; (2) increased expression of M-Ras, which is involved in bone formation; and (3) decreased expression of RASA2, which inhibits the Ras pathway and can induce MSC differentiation towards osteoblasts and promote osteogenesis (90).

## Ras family signaling in the pathogenesis of rheumatoid arthritis

RA is a persistent inflammatory disease, the common features of which include pain in joints, swelling, and irreversible damage to cartilage, tendons, and bones (91). T lymphocytes have critical roles in RA pathogenesis: they contribute to (1) autoantibody production by B lymphocytes including antibodies to citrullinated protein antigens (ACPAs) and autoantibodies against IgG Fc (92), (2) adjustment of fibroblast-like synoviocyte (FLS) activation through cell–cell interaction, (3) promotion of inflammatory cytokine production including IL-15, IL-8, and TNF-α by FLSs (91), (4) regulation of monocyte/macrophage activation, and (5) TNF-α production and osteoclast formation (93–95). Th17 cells are the most important subsets of T lymphocytes in RA patients. An increased number of this subset has been reported in the RA joints that produce IL-17 and promote the secretion of other inflammatory cytokines by synovial cells (96, 97). Furthermore, overexpression of K-Ras and its mediator B-Raf and increased phosphorylation of ERK1/2 in RA T cell have been reported (5, 98). Thus, it is not surprising that inhibition of Ras protein demonstrates the reduction of Th17 cells and pro-inflammatory cytokines such as IL-17A/F and IL-22 (63).

FLSs are one of the main cells in the synovial intimal lining that have a main role in the pathogenesis of RA disease by producing inflammatory cytokines and proteolytic enzymes that promote inflammation and cartilage destruction (99). There is more evidence that Ras proteins induce the activation of FLSs and have a role in the immune pathogenesis of RA (16). Increased expression of H-Ras in RA-FLSs has been reported, which is due to RasGRP1overexpression (100). In addition, it has been indicated that RasGRP1 contributes to the production of MMP3 and the transformed phenotype of FLSs (101).

**Figure 2:**
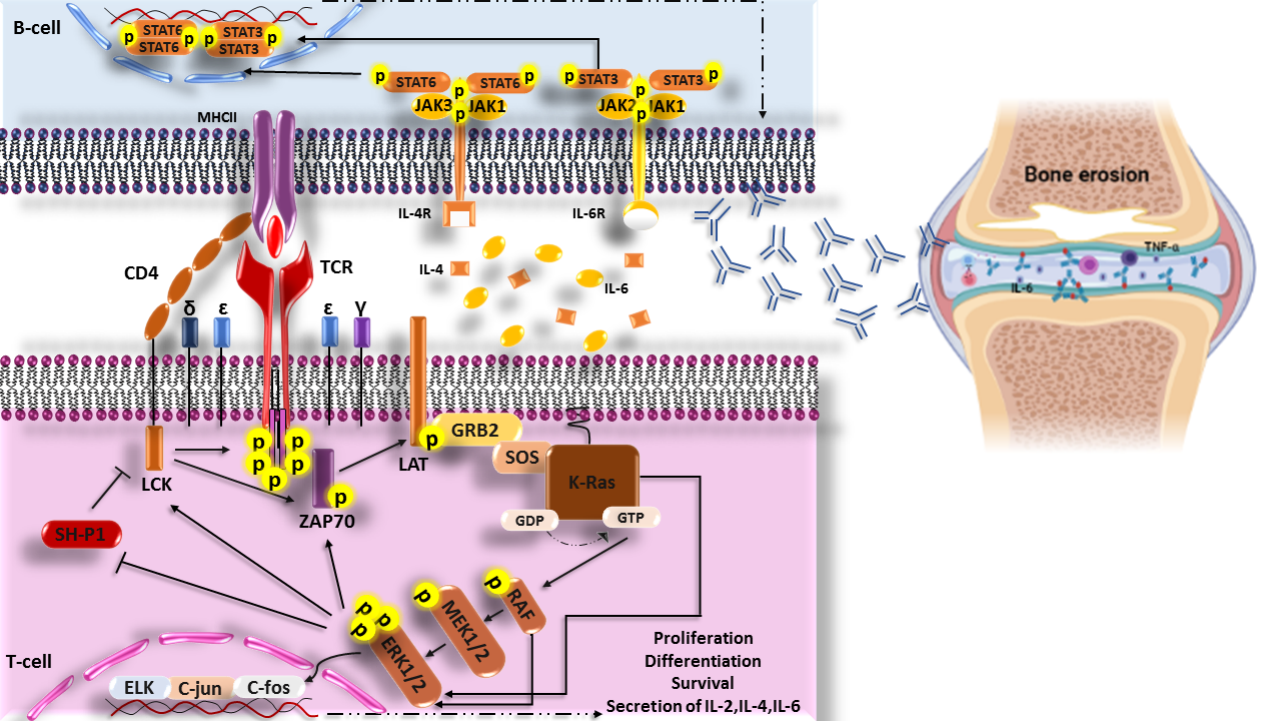
Scheme of the increased Ras signaling pathway in RA disease. Increased phosphorylation of ERK1/2 (p-ERK1/2) in T cells of RA patient’s cause’s responsiveness enhancement of T cells to neo-antigen (citrullinated proteins) and helps the production of auto-antibodies through secretin of cytokines. These auto-antibodies in joints form the immune complexes and then activate complement, immune cells, and secretions of inflammatory cytokines such as IL-6 and TNF-α that are hall markers of RA disease and finally lead to bone erosion. LCK, lymphocyte-specific protein tyrosine kinase; CD4, cluster of differentiation 4; TCR, The T-cell receptor; MHCII, Major histocompatibility complex class II molecules; ZAP70, Zeta-chain-associated protein kinase 70; LAT, linker of activated T cells; GRB2, Growth factor receptor-bound protein 2; MEK, Mitogen-activated protein kinase kinase; ERK, extracellular signal-regulated kinase; SH-P1, Src homology region 2 (SH-2) domain-containing phosphatase 1.

Interestingly, it has been revealed that transfection of FLSs with a dominant-negative mutant related to the *ras* gene in RA patients leads to the inhibition of Ras signaling, which play a critical role in decreased ERK1/2 activation, proliferation, and/or activation of FLSs, synovial hyperplasia, inflammatory cell infiltration, and high production of IL-6, which are involved in joint damage (102). In addition, suppressing the expression of c-Raf-1 (MAP kinase activator) and c-Myc (one of the c-Raf-1 downstream transcription factors) alone does not affect proliferation, morphologic appearance, or apoptosis of FLSs, but the disabling expression of both of them (c-Raf-1 and c-Myc) together induces apoptosis in FLSs. Furthermore, Triptolide, a natural compound with immunosuppressive and anti-inflammatory activities, can cause anti-proliferative effects and apoptosis in RA-FLSs through suppression of Ras-MAPK signaling (**Figure 2**) (103).

Some studies using FTI (Farnesyl transferase inhibitors), as an inhibitor of Ras protein, have shown a decrease in the severity and incidence of collagen-induced arthritis (CIA) in mice, a decrease in the expression of inflammatory cytokines such as TNF-α and IL-6 in the synovial, and a decrease in the production of TNF-induced MMP-1 by FLSs (100, 104, 105).

Osteogenesis in RA is related to enhanced osteoclast differentiation (106). Initially, pre-osteoclasts differentiate from monocyte/macrophage lineage cells under the influence of various growth factors and cytokines that are upregulated in the cell’s microenvironment, especially M-CSF and RANKL, increasing the Ras-Raf interaction and following the activation of the MEK-ERK1/2 signaling cascade. GM-CSF in mononuclear pre-osteoclasts that express upregulated GM-CSF receptor-alpha by RANKL, through the activation of Ras-ERK1/2 pathway, enhances proliferation, survival, perfusion of pre-osteoclasts, and formation of activated and multinucleated osteoclasts (68, 70).

Given the role of Ras signaling in inflammatory cytokine production, induction of osteoclast differentiation, and enhancement of FLS proliferation and activation, it seems that the Ras family signaling plays a major role in the development and progression of RA, and blockage of this signaling may be a promising strategy for RA treatment.

## Ras family signaling in systemic lupus erythematosus

SLE is an autoimmune disease that is determined by the production of autoantibodies against nuclear components, the formation of immune complex, and its deposition in different tissues such as the kidneys (6, 107). SLE’s clinical features are mainly related to B cells by losing B-lymphocyte tolerance, presentation of autoantigens to T cells and activating them, and secretion of pro-inflammatory cytokines (108). Loss of B-lymphocyte tolerance causes the autoantibody production against self-antigens and finally tissue damage. Many critical abnormalities in central tolerance have been indicated in SLE that lead to the production of autoantibodies, such as deficiency in adequate negative selection of auto-reactive B lymphocytes and insufficient receptor editing, which are critical steps in maintaining tolerance to self. On the other hand, defects in T cells can also contribute to the loss of B-cell tolerance and autoantibody production (109–112). It has been revealed that the continuous presence of B lymphocytes is related to weak curative response, and early repopulation of memory plasma blasts and B lymphocytes is associated with primary disease relapse. Moreover, selective depletion of memory B lymphocytes may reduce the risk of later renal relapse (113, 114). The numbers of plasma cells and memory B lymphocytes are enhanced in SLE patients, while naive B lymphocytes are reduced. The increased memory B cells in SLE are mature, antigen-experienced class-switched memory B cells. These cells have a lower threshold than naive cells for activation and are resistant to regulatory and inhibitory signals (114). With regard to the increased number of memory B cells in SLE and given that Ras activation leads to prolonged survival of memory B cells, it seems that Ras activation in memory B cells leads to an augmented population of these cells in SLE.

Some studies have demonstrated that Ras may correlate with disease activity in SLE. Overall, levels of Ras expression in lymphocyte cells were similar in patients with active and inactive status, but patients with the active form of the disease have shown significantly lower Ras activity (115, 116). The patient’s T cells with inactive SLE express significantly lower levels of SOS compared with healthy ones, which indicates that SOS-mediated Ras activation was restricted (115). Furthermore, the SOS in SLE T lymphocytes is not able to translocate to membrane compartments and the coupling to Grb2 protein (117). RasGRP1 is extremely expressed in T lymphocytes and is more substantial than the Grb2/SOS cascade to activate Ras; in a certain way, knocking down the RasGRP1 gene leads to the lack of both GTP-bound Ras and p-ERK1/2, although the SOS is sufficient (75). Interestingly, a spontaneous RasGRP1-disrupted mouse developed a lymph proliferative autoimmune inflammatory syndrome with a lupus-like aspect, such as anti-nuclear antibodies (ANA), anti-Smith antibodies (ASA), and anti-double-stranded DNA antibodies, splenomegaly, lymphadenopathy, and diffuse proliferative glomerulonephritis (75). In addition, single-gene mutations in RasGRP1 have been reported in SLE animal models (120). RasGRP1 impairment in T lymphocytes leads to an intense reduction in Ras-ERK1/2 signaling, disrupted in the positive selection, and the development of double-positive (CD4^+^CD8^+^) T cells (but not the negative selection) in the thymus (121). RasGRP3 and RasGRP1 play a main role in Ras-ERK1/2 signaling. The RasGRP3 expression increases in SLE B lymphocytes and PBMCs, which results in enhanced activation of B lymphocytes through ERK1/2 and AKT signaling pathways and the overproduction of inflammatory interleukins such as TNF-α and IL-6 (122). As a result, the downregulation of RasGRP1 and overexpression of RasGRP3 in the lymphocytes are related to the susceptibility of SLE. The defective Ras-ERK1/2 signaling in SLE T lymphocytes leads to reduced transcriptional mediator expression including c-FOS, c-JUN, and AP-1 (123). SLE T cells express diminished nuclear translocation of the AP-1. This impaired AP-1 signaling results in the repressed secretion of IL-2 in SLE T cells and may lead to the development of auto-reactive T lymphocytes (117). In general, decreased Ras-ERK1/2 pathway in T cells probably promotes SLE disease through decreasing DNA methylation, gene dysregulation, and auto-reactivity. Moreover, stimulation of Ras signaling in B cells results in breaking B-cell tolerance, increased number of memory B cells and plasma cells, and autoantibody production.

## Conclusion

Recently studies have revealed that Ras-ERKs are affected in the pathogenesis of auto-inflammatory rheumatic diseases. These studies indicate the harmful function of Ras-ERKs in rheumatic diseases. The activation and phosphorylation of Ras-ERKs in different cell types, including fibroblasts, osteocytes, synoviocytes, and immunocytes, can elevate disorder progression both *in vitro* and *in vivo*. Multiple studies have shown the contradictory roles of Ras-ERKs in rheumatic diseases, thus demonstrating that their outcomes can be different and context-dependent on diseases. Therefore, targeting and inhibition of factors involved in the Ras-ERK1/2 signaling pathway can be considered a key therapeutic approach for these diseases.

## Author contributions

MSS and MR searched the literature, prepared the figures, and drafted the review. MR contributed and edited the review. EF and MM drafted the review and directed the work. All authors reviewed and approved the final review.

## References

[1] Wennerberg K Rossman KL Der CJ. The ras superfamily at a glance. J Cell Sci (2005) 118(5):843–6. doi: 10.1242/jcs.01660 15731001

[2] Longnecker DS Terhune PG. What is the true rate of K-ras mutation in carcinoma of the pancreas? Pancreas (1998) 17(4):323–4. doi: 10.1097/00006676-199811000-00001 9821172

[3] Oliveira JB Bidère N Niemela JE Zheng L Sakai K Nix CP. NRAS mutation causes a human autoimmune lymphoproliferative syndrome. Proc Natl Acad Sci (2007) 104(21):8953–8. doi: 10.1073/pnas.0702975104 PMC1885609 17517660

[4] Smaldone S Olivieri J Gusella GL Moroncini G Gabrielli A Ramirez F. Ha-ras stabilization mediates pro-fibrotic signals in dermal fibroblasts. Fibrogenesis Tissue Repair (2011) 4(1):1–10. doi: 10.1186/1755-1536-4-8 21362163 PMC3059295

[5] Singh K Deshpande P Pryshchep S Colmegna I Liarski V Weyand CM. ERK-dependent T cell receptor threshold calibration in rheumatoid arthritis. J Immunol (2009) 183(12):8258–67. doi: 10.4049/jimmunol.0901784 PMC2828269 20007589

[6] Gorelik G Richardson B. Key role of ERK pathway signaling in lupus. Autoimmunity (2010) 43(1):17–22. doi: 10.3109/08916930903374832 19961364 PMC2819407

[7] Pennanen P Kallionpää RA Peltonen S Nissinen L Kähäri VM Heervä E. Signaling pathways in human osteoclasts differentiation: ERK1/2 as a key player. Mol Biol Rep (2021) 48(2):1243–54. doi: 10.1007/s11033-020-06128-5 PMC7925492 33486672

[8] Zheng G Xie Z Wang P Li J Li M Cen S. Enhanced osteogenic differentiation of mesenchymal stem cells in ankylosing spondylitis: a study based on a three-dimensional biomimetic environment. Cell Death Dis (2019) 10(5):350. doi: 10.1038/s41419-019-1586-1 31024000 PMC6484086

[9] Papaioannou G Mirzamohammadi F Kobayashi T. Ras signaling regulates osteoprogenitor cell proliferation and bone formation. Cell Death Dis (2016) 7(10):e2405. doi: 10.1038/cddis.2016.314 27735946 PMC5133981

[10] Herrmann C. Ras–effector interactions: after one decade. Curr Opin Struct Biol (2003) 13(1):122–9. doi: 10.1016/S0959-440X(02)00007-6 12581669

[11] Maruta H. PAKs, RAC/CDC42 (p21)-activated kinases: towards the cure of cancer and other PAK-dependent diseases: newnes. Melbourne, Australia: Elsevier (2013).

[12] Stokoe D Macdonald SG Cadwallader K Symons M Hancock JF. Activation of raf as a result of recruitment to the plasma membrane. Science (1994) 264(5164):1463–7. doi: 10.1126/science.7811320 7811320

[13] Leevers SJ Paterson HF Marshall CJ. Requirement for ras in raf activation is overcome by targeting raf to the plasma membrane. Nature (1994) 369(6479):411–4. doi: 10.1038/369411a0 8196769

[14] Khosravi-Far R Campbell S Rossman KL Der CJ. Increasing complexity of ras signal transduction: involvement of rho family proteins. Adv Cancer Res (1997) 72:57–107. doi: 10.1016/S0065-230X(08)60700-9 9338074

[15] Rokni M Sadeghi Shaker M Kavosi H Shokoofi S Mahmoudi M Farhadi E. The role of endothelin and RAS/ERK signaling in immunopathogenesis-related fibrosis in patients with systemic sclerosis: an updated review with therapeutic implications. Arthritis Res Ther (2022) 24(1):108. doi: 10.1186/s13075-022-02787-w 35562771 PMC9102675

[16] Reedquist KA Tak PP. Suppl 2: signal transduction pathways in chronic inflammatory autoimmune disease: small GTPases. Open Rheumatol J (2012) 6:259. doi: 10.2174/1874312901206010259 23028410 PMC3460313

[17] Hsu CL Kikuchi K Kondo M. Activation of mitogen-activated protein kinase kinase (MEK)/extracellular signal regulated kinase (ERK) signaling pathway is involved in myeloid lineage commitment. Blood (2007) 110(5):1420–8. doi: 10.1182/blood-2007-02-071761 PMC1975832 17536016

[18] Ma WT Gao F Gu K Chen DK. The role of monocytes and macrophages in autoimmune diseases: a comprehensive review. Front Immunol (2019) 10:1140. doi: 10.3389/fimmu.2019.01140 31178867 PMC6543461

[19] Navegantes KC de Souza Gomes R Pereira PAT Czaikoski PG Azevedo CHM Monteiro MC. Immune modulation of some autoimmune diseases: the critical role of macrophages and neutrophils in the innate and adaptive immunity. J Trans Med (2017) 15(1):36. doi: 10.1186/s12967-017-1141-8 PMC5312441 28202039

[20] Malyshev I Malyshev Y. Current concept and update of the macrophage plasticity concept: intracellular mechanisms of reprogramming and M3 macrophage "Switch" phenotype. BioMed Res Int (2015) 2015:341308. doi: 10.1155/2015/341308 26366410 PMC4561113

[21] He L Jhong JH Chen Q Huang KY Strittmatter K Kreuzer J. Global characterization of macrophage polarization mechanisms and identification of M2-type polarization inhibitors. Cell Rep (2021) 37(5):109955. doi: 10.1016/j.celrep.2021.109955 34731634 PMC8783961

[22] Riegel K Rajalingam K. The non-linearity of RAF-MEK signaling in dendritic cells. Cell Cycle (Georgetown Tex) (2020) 19(18):2249–59. doi: 10.1080/15384101.2020.1795990 PMC7513879 32752922

[23] van de Laar L Coffer PJ Woltman AM. Regulation of dendritic cell development by GM-CSF: molecular control and implications for immune homeostasis and therapy. Blood (2012) 119(15):3383–93. doi: 10.1182/blood-2011-11-370130 22323450

[24] Ray A Basu S Miller NM Chan AM Dittel BN. An increase in tolerogenic dendritic cell and natural regulatory T cell numbers during experimental autoimmune encephalomyelitis in rras–/– mice results in attenuated disease. J Immunol (2014) 192(11):5109–17. doi: 10.4049/jimmunol.1302254 PMC4041102 24771856

[25] Singh G Hashimoto D Yan X Helft J Park PJ Ma G. R-ras is required for murine dendritic cell maturation and CD4+ T-cell priming. Blood (2012) 119(7):1693–701. doi: 10.1182/blood-2011-05-357319 PMC3286347 22174156

[26] Kuwabara T Matsui Y Ishikawa F Kondo M. Regulation of T-cell signaling by post-translational modifications in autoimmune disease. Int J Mol Sci (2018) 19(3):819. doi: 10.3390/ijms19030819 29534522 PMC5877680

[27] McNeil LK Starr TK Hogquist KA. A requirement for sustained ERK signaling during thymocyte positive selection in vivo. Proc Natl Acad Sci (2005) 102(38):13574–9. doi: 10.1073/pnas.0505110102 PMC1224638 16174747

[28] Martelli MP Lin H Zhang W Samelson LE Bierer BE. Signaling *via* LAT (linker for T-cell activation) and Syk/ZAP70 is required for ERK activation and NFAT transcriptional activation following CD2 stimulation. Blood J Am Soc Hematol (2000) 96(6):2181–90. doi: 10.1182/blood.V96.6.2181 10979964

[29] So E-Y Oh J Jang J-Y Kim J-H Lee C-E. Ras/Erk pathway positively regulates Jak1/STAT6 activity and IL-4 gene expression in jurkat T cells. Mol Immunol (2007) 44(13):3416–26. doi: 10.1016/j.molimm.2007.02.022 17433443

[30] Baars MJ Douma T Simeonov DR Myers DR Kulhanek K Banerjee S. Dysregulated RASGRP1 expression through RUNX1 mediated transcription promotes autoimmunity. Eur J Immunol (2021) 51(2):471–82. doi: 10.1002/eji.201948451 PMC7894479 33065764

[31] Donahue AC Fruman DA. Distinct signaling mechanisms activate the target of rapamycin in response to different b-cell stimuli. Eur J Immunol (2007) 37(10):2923–36. doi: 10.1002/eji.200737281 17724683

[32] Olivieri J Coluzzi S Attolico I Olivieri A. Tirosin kinase inhibitors in chronic graft versus host disease: from bench to bedside. TheScientific World J (2011) 11:1908–31. doi: 10.1100/2011/924954 PMC3217614 22125447

[33] Krzyzowska M Swiatek W Fijalkowska B Niemialtowski M Schollenberger A. The role of MAP kinases in immune response. Adv Cell Biol (2010) 2:125–38. doi: 10.2478/v10052-010-0007-5

[34] Teodorovic LS Babolin C Rowland SL Greaves SA Baldwin DP Torres RM. Activation of ras overcomes b-cell tolerance to promote differentiation of autoreactive b cells and production of autoantibodies. Proc Natl Acad Sci (2014) 111(27):E2797–E806. doi: 10.1073/pnas.1402159111 PMC4103347 24958853

[35] Deng G-M Kyttaris VC Tsokos GC. Targeting syk in autoimmune rheumatic diseases. Front Immunol (2016) 7. doi: 10.3389/fimmu.2016.00078 PMC4779881 27014261

[36] Greaves SA Peterson JN Torres RM Pelanda R. Activation of the MEK-ERK pathway is necessary but not sufficient for breaking central b cell tolerance. Front Immunol (2018) 9:707. doi: 10.3389/fimmu.2018.00707 29686680 PMC5900439

[37] Takahashi Y Inamine A Hashimoto S Haraguchi S Yoshioka E Kojima N. Novel role of the ras cascade in memory b cell response. Immunity (2005) 23(2):127–38. doi: 10.1016/j.immuni.2005.06.010 16111632

[38] Vanshylla K Bartsch C Hitzing C Krümpelmann L Wienands J Engels N. Grb2 and GRAP connect the b cell antigen receptor to erk MAP kinase activation in human b cells. Sci Rep (2018) 8(1):4244. doi: 10.1038/s41598-018-22544-x 29523808 PMC5844867

[39] Coughlin JJ Stang SL Dower NA Stone JC. RasGRP1 and RasGRP3 regulate b cell proliferation by facilitating b cell receptor-ras signaling. J Immunol (2005) 175(11):7179–84. doi: 10.4049/jimmunol.175.11.7179 16301621

[40] Fuentes-Calvo I Blázquez-Medela AM Eleno N Santos E López-Novoa JM Martínez-Salgado C. H-ras isoform modulates extracellular matrix synthesis, proliferation, and migration in fibroblasts. Am J Physiology-Cell Physiol (2012) 302(4):C686–C97. doi: 10.1152/ajpcell.00103.2011 22094331

[41] López-Novoa JM Nieto MA. Inflammation and EMT: an alliance towards organ fibrosis and cancer progression. EMBO Mol Med (2009) 1(6-7):303–14. doi: 10.1002/emmm.200900043 PMC3378143 20049734

[42] Kim H Choi JA Kim JH. Ras promotes transforming growth factor-β (TGF-β)-induced epithelial-mesenchymal transition *via* a leukotriene B4 receptor-2-linked cascade in mammary epithelial cells. J Biol Chem (2014) 289(32):22151–60. doi: 10.1074/jbc.M114.556126 PMC4139228 24990945

[43] Sharpe CC Dockrell ME Noor MI Monia BP Hendry BM. Role of ras isoforms in the stimulated proliferation of human renal fibroblasts in primary culture. J Am Soc Nephrology (2000) 11(9):1600–6. doi: 10.1681/ASN.V1191600 10966484

[44] Tripathi K Garg M. Mechanistic regulation of epithelial-to-mesenchymal transition through RAS signaling pathway and therapeutic implications in human cancer. J Cell Communication Signaling (2018) 12(3):513–27. doi: 10.1007/s12079-017-0441-3 PMC6039341 29330773

[45] Santamaria PG Nebreda AR. Deconstructing ERK signaling in tumorigenesis. Mol Cell (2010) 38(1):3–5. doi: 10.1016/j.molcel.2010.03.012 20385084

[46] Zuo JH Zhu W Li MY Li XH Yi H Zeng GQ. Activation of EGFR promotes squamous carcinoma SCC10A cell migration and invasion *via* inducing EMT-like phenotype change and MMP-9-mediated degradation of e-cadherin. J Cell Biochem (2011) 112(9):2508–17. doi: 10.1002/jcb.23175 21557297

[47] Thiery JP. Cell adhesion in development: a complex signaling network. Curr Opin Genet Dev (2003) 13(4):365–71. doi: 10.1016/S0959-437X(03)00088-1 12888009

[48] Finnson KW Almadani Y Philip A. Non-canonical (non-SMAD2/3) TGF-β signaling in fibrosis: mechanisms and targets. In seminars in cell & developmental biology. Academic Press. (2020) 101:115–122 10.1016/j.semcdb.2019.11.013 31883994

[49] Higgins SP Tang Y Higgins CE Mian B Zhang W Czekay R-P. TGF-β1/p53 signaling in renal fibrogenesis. Cell Signalling (2018) 43:1–10. doi: 10.1016/j.cellsig.2017.11.005 29191563 PMC5860677

[50] Reich N Maurer B Akhmetshina A Venalis P Dees C Zerr P. The transcription factor fra-2 regulates the production of extracellular matrix in systemic sclerosis. Arthritis Rheumatism: Off J Am Coll Rheumatol (2010) 62(1):280–90. doi: 10.1002/art.25056 20039427

[51] Gabrielli A Svegliati S Moroncini G Amico D. Suppl 1: new insights into the role of oxidative stress in scleroderma fibrosis. Open Rheumatol J (2012) 6:87. doi: 10.2174/1874312901206010087 22802906 PMC3395898

[52] Richter K Kietzmann T. Reactive oxygen species and fibrosis: further evidence of a significant liaison. Cell Tissue Res (2016) 365(3):591–605. doi: 10.1007/s00441-016-2445-3 27345301 PMC5010605

[53] Jobling MF Mott JD Finnegan MT Jurukovski V Erickson AC Walian PJ. Isoform-specific activation of latent transforming growth factor β (LTGF-β) by reactive oxygen species. Radiat Res (2006) 166(6):839–48. doi: 10.1667/RR0695.1 17149983

[54] Dosoki H. The role of NADPH oxidase in the pathogenesis of systemic sclerosis. Münster, Germany: Universitäts-und Landesbibliothek Münster (2016).

[55] Piera-Velazquez S Jimenez SA. Role of cellular senescence and NOX4-mediated oxidative stress in systemic sclerosis pathogenesis. Curr Rheumatol Rep (2015) 17(1):1–11. doi: 10.1007/s11926-014-0473-0 PMC6779060 25475596

[56] Svegliati S Cancello R Sambo P Luchetti M Paroncini P Orlandini G. Platelet-derived growth factor and reactive oxygen species (ROS) regulate ras protein levels in primary human fibroblasts *via* ERK1/2: amplification of ROS and ras in systemic sclerosis fibroblasts. J Biol Chem (2005) 280(43):36474–82. doi: 10.1074/jbc.M502851200 16081426

[57] Park M Kim M Suh Y Kim R Kim H Lim E. Novel signaling axis for ROS generation during K-ras-induced cellular transformation. Cell Death Differentiation (2014) 21(8):1185–97. doi: 10.1038/cdd.2014.34 PMC4085525 24632950

[58] Andrae J Gallini R Betsholtz C. Role of platelet-derived growth factors in physiology and medicine. Genes Dev (2008) 22(10):1276–312. doi: 10.1101/gad.1653708 PMC2732412 18483217

[59] Svegliati Baroni S Santillo M Bevilacqua F Luchetti M Spadoni T Mancini M. Stimulatory autoantibodies to the PDGF receptor in systemic sclerosis. New Engl J Med (2006) 354(25):2667–76. doi: 10.1056/NEJMoa052955 16790699

[60] Bonner JC. Regulation of PDGF and its receptors in fibrotic diseases. Cytokine Growth factor Rev (2004) 15(4):255–73. doi: 10.1016/j.cytogfr.2004.03.006 15207816

[61] Ancrile BB O'Hayer KM Counter CM. Oncogenic ras-induced expression of cytokines: a new target of anti-cancer therapeutics. Mol Interventions (2008) 8(1):22–7. doi: 10.1124/mi.8.1.6 PMC2788125 18332481

[62] Lin F Zhu Y Hu G. Naringin promotes cellular chemokine synthesis and potentiates mesenchymal stromal cell migration *via* the ras signaling pathway. Exp Ther Med (2018) 16(4):3504–10. doi: 10.3892/etm.2018.6634 PMC6143896 30233702

[63] Zayoud M Marcu-Malina V Vax E Jacob-Hirsch J Elad-Sfadia G Barshack I. Ras signaling inhibitors attenuate disease in adjuvant-induced arthritis *via* targeting pathogenic antigen-specific Th17-type cells. Front Immunol (2017) 8:799. doi: 10.3389/fimmu.2017.00799 28736556 PMC5500629

[64] Fujii H. Mechanisms of signal transduction from receptors of type I and type II cytokines. J immunotoxicology (2007) 4(1):69–76. doi: 10.1080/15476910601154779 18958714

[65] Kinbara K Goldfinger LE Hansen M Chou FL Ginsberg MH. Ras GTPases: integrins' friends or foes? Nat Rev Mol Cell Biol (2003) 4(10):767–76. doi: 10.1038/nrm1229 14570053

[66] Xu XS Vanderziel C Bennett CF Monia BP. A role for c-raf kinase and ha-ras in cytokine-mediated induction of cell adhesion molecules. J Biol Chem (1998) 273(50):33230–8. doi: 10.1074/jbc.273.50.33230 9837893

[67] Lee K Chung YH Ahn H Kim H Rho J Jeong D. Selective regulation of MAPK signaling mediates RANKL-dependent osteoclast differentiation. Int J Biol Sci (2016) 12(2):235. doi: 10.7150/ijbs.13814 26884720 PMC4737679

[68] Lee K Seo I Choi MH Jeong D. Roles of mitogen-activated protein kinases in osteoclast biology. Int J Mol Sci (2018) 19(10):3004. doi: 10.3390/ijms19103004 30275408 PMC6213329

[69] Jiang B Yuan C Han J Shen M Zhou X Zhou L. miR-143-3p inhibits the differentiation of osteoclast induced by synovial fibroblast and monocyte coculture in adjuvant-induced arthritic rats. BioMed Res Int (2021) 2021. doi: 10.1155/2021/5565973 PMC8416385 34485516

[70] Lee MS Kim HS Yeon J-T Choi S-W Chun CH Kwak HB. GM-CSF regulates fusion of mononuclear osteoclasts into bone-resorbing osteoclasts by activating the Ras/ERK pathway. J Immunol (2009) 183(5):3390–9. doi: 10.4049/jimmunol.0804314 19641137

[71] Weivoda MM Oursler MJ. The roles of small GTPases in osteoclast biology. Orthopedic muscular system Curr Res (2014) 3. doi: 10.4172/2161-0533.1000161 PMC4296324 25599004

[72] Schindeler A Little DG. Ras-MAPK signaling in osteogenic differentiation: friend or foe? J Bone mineral Res Off J Am Soc Bone Mineral Res (2006) 21(9):1331–8. doi: 10.1359/jbmr.060603 16939391

[73] Gabrielli A Avvedimento EV Krieg T. Scleroderma. N Engl J Med (2009) 360(19):1989–2003. doi: 10.1056/NEJMra0806188 19420368

[74] Sierra-Sepúlveda A Esquinca-González A Benavides-Suárez SA Sordo-Lima DE Caballero-Islas AE Cabral-Castañeda AR. Systemic sclerosis pathogenesis and emerging therapies, beyond the fibroblast. BioMed Res Int (2019) 2019. doi: 10.1155/2019/4569826 PMC6364098 30809542

[75] Stone J. C.. Regulation of Ras in lymphocytes: get a GRP. Biochem Soc Trans (2006) 34(5):858–61. doi: 10.1042/BST0340858 17052215

[76] Roumm AD Whiteside TL Medsger TA Jr. Rodnan GP. Lymphocytes in the skin of patients with progressive systemic sclerosis. Arthritis Rheumatism: Off J Am Coll Rheumatol (1984) 27(6):645–53. doi: 10.1002/art.1780270607 6375682

[77] Whitfield ML Finlay DR Murray JI Troyanskaya OG Chi J-T Pergamenschikov A. Systemic and cell type-specific gene expression patterns in scleroderma skin. Proc Natl Acad Sci (2003) 100(21):12319–24. doi: 10.1073/pnas.1635114100 PMC218756 14530402

[78] Gardner H Shearstone JR Bandaru R Crowell T Lynes M Trojanowska M. Gene profiling of scleroderma skin reveals robust signatures of disease that are imperfectly reflected in the transcript profiles of explanted fibroblasts. Arthritis Rheumatism (2006) 54(6):1961–73. doi: 10.1002/art.21894 16736506

[79] Meloni F Solari N Cavagna L Morosini M Montecucco CM Fietta AM. Frequency of Th1, Th2 and Th17 producing T lymphocytes in bronchoalveolar lavage of patients with systemic sclerosis. Clin Exp Rheumatol (2009) 27(5):765. 19917158

[80] Sakkas LI Platsoucas CD. Is systemic sclerosis an antigen-driven T cell disease? Arthritis Rheumatism: Off J Am Coll Rheumatol (2004) 50(6):1721–33. doi: 10.1002/art.20315 15188347

[81] Chizzolini C Parel Y De Luca C Tyndall A Åkesson A Scheja A. Systemic sclerosis Th2 cells inhibit collagen production by dermal fibroblasts *via* membrane-associated tumor necrosis factor α. Arthritis Rheumatism: Off J Am Coll Rheumatol (2003) 48(9):2593–604. doi: 10.1002/art.11129 13130479

[82] Mavalia C Scaletti C Romagnani P Carossino AM Pignone A Emmi L. Type 2 helper T-cell predominance and high CD30 expression in systemic sclerosis. Am J Pathol (1997) 151(6):1751. 9403725 PMC1858349

[83] O'reilly S Cant R Ciechomska M Van Laar JM. Interleukin-6: a new therapeutic target in systemic sclerosis? Clin Trans Immunol (2013) 2(4):e4. doi: 10.1038/cti.2013.2 PMC4232056 25505952

[84] Meléndez GC McLarty JL Levick SP Du Y Janicki JS Brower GL. Interleukin 6 mediates myocardial fibrosis, concentric hypertrophy, and diastolic dysfunction in rats. Hypertension (2010) 56(2):225–31. doi: 10.1161/HYPERTENSIONAHA.109.148635 PMC2921860 20606113

[85] Shao F Liu Q Zhu Y Fan Z Chen W Liu S. Targeting chondrocytes for arresting bony fusion in ankylosing spondylitis. Nat Commun (2021) 12(1):6540. doi: 10.1038/s41467-021-26750-6 34764263 PMC8585952

[86] Zhang L Li YG Li YH Qi L Liu XG Yuan CZ. Increased frequencies of Th22 cells as well as Th17 cells in the peripheral blood of patients with ankylosing spondylitis and rheumatoid arthritis. PloS One (2012) 7(4):e31000. doi: 10.1371/journal.pone.0031000 22485125 PMC3317658

[87] Holt VE Luo Y Colbert RA. IL-22 effects on osteoblast mineralization and metabolic profile. J Immunol (2020) 204(1 Supplement):73.7–.7. doi: 10.4049/jimmunol.204.Supp.73.7

[88] Li Y Wang P Xie Z Huang L Yang R Gao L. Whole genome expression profiling and signal pathway screening of MSCs in ankylosing spondylitis. Stem Cells Int (2014) 2014:913050. doi: 10.1155/2014/913050 25544849 PMC4269092

[89] Watanabe-Takano H Takano K Keduka E Endo T. M-ras is activated by bone morphogenetic protein-2 and participates in osteoblastic determination, differentiation, and transdifferentiation. Exp Cell Res (2010) 316(3):477–90. doi: 10.1016/j.yexcr.2009.09.028 19800879

[90] Young LC Rodriguez-Viciana P. MRAS: a close but understudied member of the RAS family. Cold Spring Harbor Perspect Med (2018) 8(12):a033621. doi: 10.1101/cshperspect.a033621 PMC6280710 29311130

[91] Cho ML Yoon CH Hwang SY Park MK Min SY Lee SH. Effector function of type II collagen–stimulated T cells from rheumatoid arthritis patients: cross-talk between T cells and synovial fibroblasts. Arthritis Rheumatism (2004) 50(3):776–84. doi: 10.1002/art.20106 15022319

[92] Moher D Liberati A Tetzlaff J Altman DG Group P. Reprint–preferred reporting items for systematic reviews and meta-analyses: the PRISMA statement. Phys Ther (2009) 89(9):873–80. doi: 10.1093/ptj/89.9.873 19723669

[93] McInnes IB Leung BP Sturrock RD Field M Liew FY. Interleukin-15 mediates T cell-dependent regulation of tumor necrosis factor-α production in rheumatoid arthritis. Nat Med (1997) 3(2):189–95. doi: 10.1038/nm0297-189 9018238

[94] Kotake S Udagawa N Hakoda M Mogi M Yano K Tsuda E. Activated human T cells directly induce osteoclastogenesis from human monocytes: possible role of T cells in bone destruction in rheumatoid arthritis patients. Arthritis Rheumatism: Off J Am Coll Rheumatol (2001) 44(5):1003–12. doi: 10.1002/1529-0131(200105)44:5<1003::AID-ANR179>3.0.CO;2-# 11352231

[95] Kim HR Kim KW Kim BM Jung HG Cho ML Lee SH. Reciprocal activation of CD4+ T cells and synovial fibroblasts by stromal cell–derived factor 1 promotes RANKL expression and osteoclastogenesis in rheumatoid arthritis. Arthritis Rheumatol (2014) 66(3):538–48. doi: 10.1002/art.38286 24574213

[96] Fossiez F Djossou O Chomarat P Flores-Romo L Ait-Yahia S Maat C. T Cell interleukin-17 induces stromal cells to produce proinflammatory and hematopoietic cytokines. J Exp Med (1996) 183(6):2593–603. doi: 10.1084/jem.183.6.2593 PMC2192621 8676080

[97] Li N Wang JC Liang TH Zhu MH Wang JY Fu XL. Pathologic finding of increased expression of interleukin-17 in the synovial tissue of rheumatoid arthritis patients. Int J Clin Exp Pathol (2013) 6(7):1375. 23826419 PMC3693203

[98] Singh K Deshpande P Li G Yu M Pryshchep S Cavanagh M. K-RAS GTPase- and b-RAF kinase-mediated T-cell tolerance defects in rheumatoid arthritis. Proc Natl Acad Sci USA (2012) 109(25):E1629–37. doi: 10.1073/pnas.1117640109 PMC3382540 22615393

[99] Bartok B Firestein GS. Fibroblast-like synoviocytes: key effector cells in rheumatoid arthritis. Immunol Rev (2010) 233(1):233–55. doi: 10.1111/j.0105-2896.2009.00859.x PMC2913689 20193003

[100] Abeles AM Marjanovic N Park J Attur M Chan ES Al-Mussawir HE. Protein isoprenylation regulates secretion of matrix metalloproteinase 1 from rheumatoid synovial fibroblasts: effects of statins and farnesyl and geranylgeranyl transferase inhibitors. Arthritis Rheumatism: Off J Am Coll Rheumatol (2007) 56(9):2840–53. doi: 10.1002/art.22824 17763406

[101] Abreu JR de Launay D Sanders ME Grabiec AM van de Sande MG Tak PP. The ras guanine nucleotide exchange factor RasGRF1 promotes matrix metalloproteinase-3 production in rheumatoid arthritis synovial tissue. Arthritis Res Ther (2009) 11(4):1–13. doi: 10.1186/ar2785 PMC2745805 19678938

[102] Yamamoto A Fukuda A Seto H Miyazaki T Kadono Y Sawada Y. Suppression of arthritic bone destruction by adenovirus-mediated dominant-negative ras gene transfer to synoviocytes and osteoclasts. Arthritis Rheumatism: Off J Am Coll Rheumatol (2003) 48(9):2682–92. doi: 10.1002/art.11214 13130489

[103] Zhang Q Liu J Zhang M Wei S Li R Gao Y. Apoptosis induction of fibroblast-like synoviocytes is an important molecular-mechanism for herbal medicine along with its active components in treating rheumatoid arthritis. Biomolecules (2019) 9(12):795. doi: 10.3390/biom9120795 31795133 PMC6995542

[104] Na H-J Lee S-J Kang Y-C Cho Y-L Nam W-D Kim PK. Inhibition of farnesyltransferase prevents collagen-induced arthritis by down-regulation of inflammatory gene expression through suppression of p21ras-dependent NF-κB activation. J Immunol (2004) 173(2):1276–83. doi: 10.4049/jimmunol.173.2.1276 15240720

[105] Philips MR Cox AD. Geranylgeranyltransferase I as a target for anti-cancer drugs. J Clin Invest (2007) 117(5):1223–5. doi: 10.1172/JCI32108 PMC1857249 17476354

[106] Steffen U Schett G Bozec A. How autoantibodies regulate osteoclast induced bone loss in rheumatoid arthritis. Front Immunol (2019) 10:1483. doi: 10.3389/fimmu.2019.01483 31333647 PMC6619397

[107] Sandoughi M Saravani M Rokni M Nora M Mehrabani M Dehghan A. Association between COX-2 and 15-PGDH polymorphisms and SLE susceptibility. Int J Rheumatic Dis (2020) 23(5):627–32. doi: 10.1111/1756-185X.13808 32100450

[108] Nashi E Wang Y Diamond B. The role of b cells in lupus pathogenesis. Int J Biochem Cell Biol (2010) 42(4):543–50. doi: 10.1016/j.biocel.2009.10.011 PMC2835836 19850148

[109] Choileain NN Redmond H. Regulatory T-cells and autoimmunity. J Surg Res (2006) 130(1):124–35. doi: 10.1016/j.jss.2005.07.033 16154142

[110] Blanco P Viallard J-F Pellegrin J-L Moreau J-F. Cytotoxic T lymphocytes and autoimmunity. Curr Opin Rheumatol (2005) 17(6):731–4. doi: 10.1097/01.bor.0000179942.27777.f8 16224251

[111] Jiang H Chess L. Regulation of immune responses by T cells. N Engl J Med (2006) 354(11):1166–76. doi: 10.1056/NEJMra055446 16540617

[112] La Cava A Fang CJ Singh RP Ebling F Hahn BH. Manipulation of immune regulation in systemic lupus erythematosus. Autoimmun Rev (2005) 4(8):515–9. doi: 10.1016/j.autrev.2005.04.008 16214088

[113] Yap DYH Chan TM. B cell abnormalities in systemic lupus erythematosus and lupus nephritis-role in pathogenesis and effect of immunosuppressive treatments. Int J Mol Sci (2019) 20(24):6231. doi: 10.3390/ijms20246231 31835612 PMC6940927

[114] Karrar S Cunninghame Graham DS. Abnormal b cell development in systemic lupus erythematosus: what the genetics tell us. Arthritis Rheumatol (Hoboken NJ) (2018) 70(4):496–507. doi: 10.1002/art.40396 PMC5900717 29207444

[115] Rapoport M Mor A Amit M Rosenberg R Ramot Y Mizrachi A. Decreased expression of the p21ras stimulatory factor hSOS in PBMC from inactive SLE patients. Lupus (1999) 8(1):24–8. doi: 10.1191/096120399678847362 10025596

[116] Rapoport MJ Amit M Aharoni D Weiss M Weissgarten J Bruck N. Constitutive up-regulated activity of MAP kinase is associated with down-regulated early p21Ras pathway in lymphocytes of SLE patients. J Autoimmun (2002) 19(1-2):63–70. doi: 10.1006/jaut.2002.0596 12367560

[117] Cedeño S Cifarelli DF Blasini AM Paris M Placeres F Alonso G. Defective activity of ERK-1 and ERK-2 mitogen-activated protein kinases in peripheral blood T lymphocytes from patients with systemic lupus erythematosus: potential role of altered coupling of ras guanine nucleotide exchange factor hSos to adapter protein Grb2 in lupus T cells. Clin Immunol (2003) 106(1):41–9. doi: 10.1016/S1521-6616(02)00052-9 12584050

[118] Chen YF Morel L. Genetics of T cell defects in lupus. Cell Mol Immunol (2005) 2(6):403–9. 16426489

[119] Xue C Lan-Lan W Bei C Jie C Wei-Hua F. Abnormal Fas/FasL and caspase-3-mediated apoptotic signaling pathways of T lymphocyte subset in patients with systemic lupus erythematosus. Cell Immunol (2006) 239(2):121–8. doi: 10.1016/j.cellimm.2006.05.003 16808908

[120] Marti F Garcia GG Lapinski PE MacGregor JN King PD. Essential role of the T cell–specific adapter protein in the activation of LCK in peripheral T cells. J Exp Med (2006) 203(2):281–7. doi: 10.1084/jem.20051637 PMC2118198 16446380

[121] Norment AM Bogatzki LY Klinger M Ojala EW Bevan MJ Kay RJ. Transgenic expression of RasGRP1 induces the maturation of double-negative thymocytes and enhances the production of CD8 single-positive thymocytes. J Immunol (2003) 170(3):1141–9. doi: 10.4049/jimmunol.170.3.1141 12538669

[122] An X-J Xia Y Li J Dong L-Y Wang Y-J Yang J. RasGRP3 in peripheral blood mononuclear cells is associated with disease activity and implicated in the development of systemic lupus erythematosus. Am J Trans Res (2019) 11(3):1800. PMC6456560 30972203

[123] Hickman SP Yang J Thomas RM Wells AD Turka LA. Defective activation of protein kinase c and ras-ERK pathways limits IL-2 production and proliferation by CD4+ CD25+ regulatory T cells. J Immunol (2006) 177(4):2186–94. doi: 10.4049/jimmunol.177.4.2186 16887978

